# (*E*)-3-(4-Chloro­phen­yl)-1-(1-naphth­yl)prop-2-en-1-one

**DOI:** 10.1107/S1600536809050508

**Published:** 2009-11-28

**Authors:** Grzegorz Dutkiewicz, S. Samshuddin, B. Narayana, H. S. Yathirajan, Maciej Kubicki

**Affiliations:** a Department of Chemistry, Adam Mickiewicz University, Grunwaldzka 6, 60-780 Poznań, Poland; b Department of Studies in Chemistry, Mangalore University, Mangalagangotri 574 199, India; c Department of Studies in Chemistry, University of Mysore, Mysore 570 006, India

## Abstract

In the title compound, C_19_H_13_ClO, the benzene ring and the naphthalene system, are twisted by 12.3 (3) and 36.1 (2)°, respectively, and in opposite directions with respect to the central propenone bridge. The bond-angle pattern within the benzene ring is influence by both substituents; these influences are almost additive. In the crystal, the molecules are linked by C—H⋯O and C—H⋯Cl inter­actions.

## Related literature

For chalcones, see: Dhar (1981 ▶); Di Carlo *et al.* (1999 ▶); Dimmock *et al.* (1999 ▶); Goto *et al.* (1991 ▶); Indira *et al.* (2002 ▶); Sarojini *et al.* (2006 ▶); Satyanarayana *et al.* (2004 ▶); Uchida *et al.* (1998 ▶); Yarishkin (2008 ▶). For the 1-naphtyl analogue, see: Eswaramoorthy *et al.* (1994 ▶). For the influence of substituents on the geometry of the phenyl ring, see: Domenicano (1988 ▶). For a description of the Cambridge Crystallographic Database, see: Allen (2002 ▶).
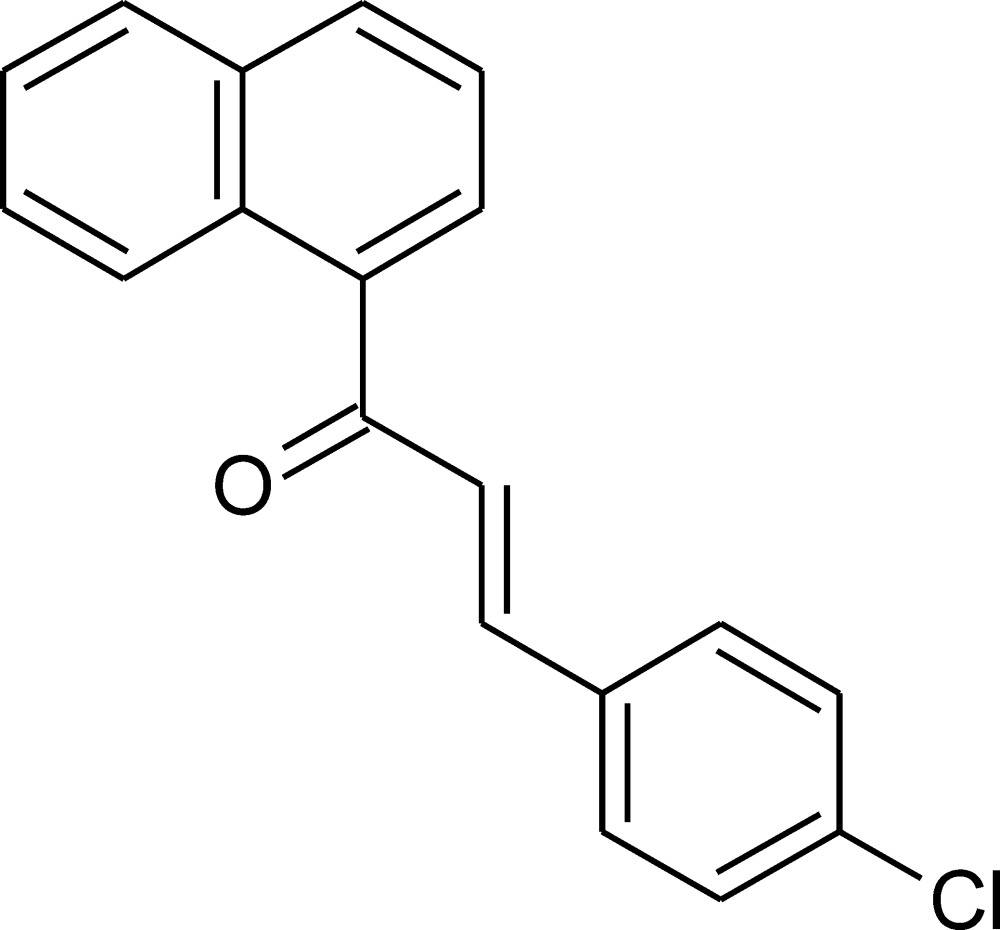


## Experimental

### 

#### Crystal data

C_19_H_13_ClO
*M* *_r_* = 292.74
Monoclinic, 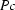

*a* = 12.0392 (14) Å
*b* = 8.0544 (5) Å
*c* = 7.8472 (6) Å
β = 98.091 (10)°
*V* = 753.36 (11) Å^3^
*Z* = 2
Mo *K*α radiation
μ = 0.25 mm^−1^
*T* = 295 K
0.30 × 0.20 × 0.15 mm

#### Data collection

Oxford Diffraction Xcalibur (Sapphire2, large Be window) diffractometer
Absorption correction: multi-scan (*CrysAlis PRO*; Oxford Diffraction, 2009 ▶) *T*_min_ = 0.666, *T*_max_ = 1.000
2481 measured reflections
1866 independent reflections
1440 reflections with *I* > 2σ(*I*)
*R*_int_ = 0.014

#### Refinement

*R*[*F* ^2^ > 2σ(*F* ^2^)] = 0.035
*wR*(*F* ^2^) = 0.083
*S* = 0.96
1866 reflections
190 parameters
2 restraints
H-atom parameters constrained
Δρ_max_ = 0.12 e Å^−3^
Δρ_min_ = −0.13 e Å^−3^
Absolute structure: Flack (1983 ▶), 423 Friedel pairs
Flack parameter: 0.08 (7)

### 

Data collection: *CrysAlis PRO* (Oxford Diffraction, 2009 ▶); cell refinement: *CrysAlis PRO*; data reduction: *CrysAlis PRO*; program(s) used to solve structure: *SIR92* (Altomare *et al.*, 1993 ▶); program(s) used to refine structure: *SHELXL97* (Sheldrick, 2008 ▶); molecular graphics: *Stereochemical Workstation Operation Manual* (Siemens, 1989 ▶); software used to prepare material for publication: *SHELXL97*.

## Supplementary Material

Crystal structure: contains datablocks I, global. DOI: 10.1107/S1600536809050508/fk2008sup1.cif

Structure factors: contains datablocks I. DOI: 10.1107/S1600536809050508/fk2008Isup2.hkl

Additional supplementary materials:  crystallographic information; 3D view; checkCIF report

## Figures and Tables

**Table 1:** Hydrogen-bond geometry (Å, °)

*D*—H⋯*A*	*D*—H	H⋯*A*	*D*⋯*A*	*D*—H⋯*A*
C17—H17⋯O12^i^	0.93	2.42	3.180 (4)	139
C3—H3⋯Cl21^ii^	0.93	2.92	3.703 (3)	143
C8—H8⋯O12^iii^	0.93	2.64	3.546 (4)	163

Symmetry codes: (i) 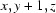; (ii) 
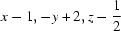
; (iii) 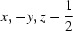.

**Fig. 1.**
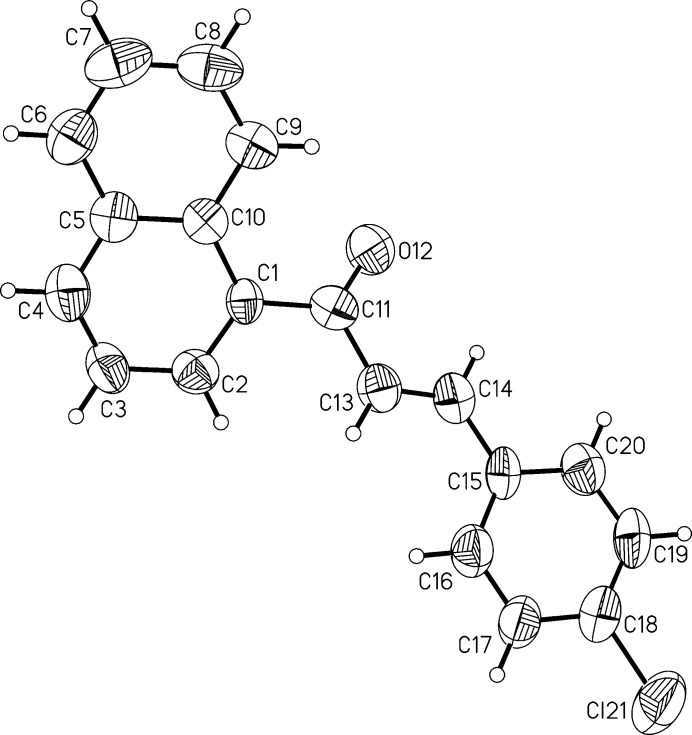
Anisotropic ellipsoid representation of the compound I together with atom labelling scheme. The ellipsoids are drawn at 50% probability level, hydrogen atoms are depicted as spheres with arbitrary radii.

**Fig. 2.**
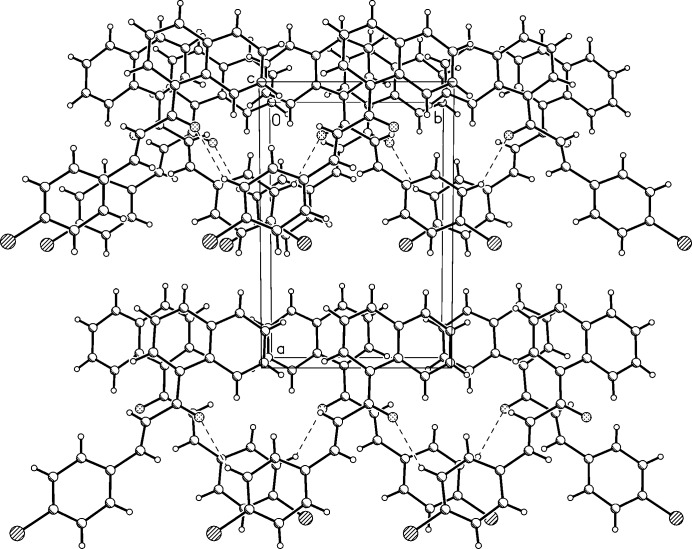
The crystal packing of I as seen along z direction; weak C—H···O bonds are shown as dashed lines.

**Fig. 3.**
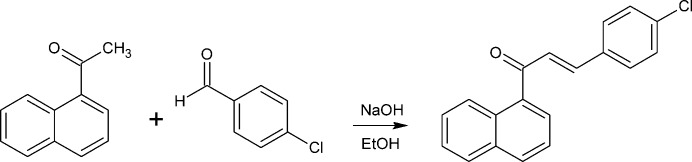
The formation of the title compound.

## supplementary crystallographic information

### Comment

Chalcones constitute an important family of flavonoids. They have been reported to possess many interesting pharmacological activities (Dhar, 1981) including anti-inflammatory, antimicrobial, antifungal, antioxidant, cytotoxic, antitumor and anticancer activities (Dimmock *et al.*, 1999; Satyanarayana *et al.*, 2004). Some chalcones demonstrated the ability to block voltage-dependent potassium channels (Yarishkin *et al.*, 2008). Chalcones are also finding application as organic nonlinear optical materials (NLO) for their SHG conversion efficiency (Sarojini *et al.*, 2006). Chalcone derivatives are recognized material in the NLO applications because of their excellent blue light transmittance and good crystallization ability (Goto *et al.*,1991; Uchida *et al.*,1998; Indira *et al.*, 2002). Chemically chalcones consists of open-chain flavonoids in which the two aromatic rings are joined by α,\&s-unsaturated carbonyl system. The radical quenching properties of the phenolic groups present in many chalcones have raised interest in using these compounds or chalcone rich plant extracts as drugs or food preservatives (Di Carlo *et al.*, 1999). As a part of our efforts on the synthesis of naphthyl chalcones, this paper describes the crystal structure of a new naphthyl chalcone, (2*E*)-3-(4-Chlorophenyl)-1-(naphthalen-1-yl)prop-2-en-1-one (I, Scheme 1). There are 298 structures in the Cambridge Crystallographic Database (Allen, 2002: Ver. 5.30, Nov. 2008, last update Sep. 2009) that posses two aromatic moieties connected *via* CH=CH—CO– fragment, but only 10 of them are naphthyl chalcones, and the single one example with 1-naphthalene substituent (1-(1-Naphthalenyl)-3-(4-nitrophenyl)-2-propenone, Eswaramoorthy *et al.*, 1994). It might be noted, that this structure apparently has errors: one of the torsion angles in the aromatic ring is as large as 5°.

The molecule of I is built of three approximately planar fragments (Fig. 1): the phenyl ring (A, maximum deviation form the least-squares plane is 0.006 (3) Å), propenone fragment C=C—C=O (B, 0.014 (2) Å), and the naphtalene ring system (C). This last fragment however is significantly folded, even though both individual rings are almost planar, the dihedral angle between these planes is as high as 5.05 (13)°. The overall conformation of I can be described in terms of dihedral angles between these fragments: A/B 36.1 (2)°, B/C 12.3 (3)°, and A/C 25.51 (9)°. These values show that the terminal planes are twisted in opposite sense with respect to the central bridging fragment. This situation is relatively rare, the majority of chalcones found in the CDB shows the same sense of rotation with respect to the central bridge.

The bond lengths within the conjugated linear fragment suggest the large degree of localization: C—C bond length of 1.480 (4) Å, C=C of 1.329 (4)Å and C=O 1.232 (3) Å. The bond angles within the phenyl ring are influenced by both Cl and C=C– substituents, and these influences are almost additive, as suggested *e.g.*, by Domenicano (1988). The distribution is almost symmetrical with respect to the C15···C18 line, and the values are close to those calculated by summing the effects of both substituents.

In the crystal structure there is one relatively short C—H···O potential hydrogen bond, C17—H17···O12(*x*,1 + *y*,*z*), that link molecules into infinite chains along *y* direction, and few weak C—H···O and C—H···π contacts, which can stabilize the packing otherwise determined by the van der Waals interactions and close packing requirements (Fig. 2).

### Experimental

To a mixture of 1-acetonaphthone (1.7 g, 0.01 mol) and *p*-chlorobenzaldehyde (1.4 g, 0.01 mol) in 30 ml e thanol, 10 ml of 10% sodium hydroxide solution was added and stirred at 5–10 *^o^*C for 3 h. The precipitate formed was collected by filtration and purified by recrystallization from ethanol. The single-crystal was grown from DMF by slow evaporation method and yield of the compound was 82%. (m.p. 360–362 K). Analytical data: Found (Calculated): C %: 76.89 (77.95); H %: 4.23 (4.48).

### Refinement

Hydrogen atoms were located geometrically and refined as a riding model; their *U*_iso_ values were set at 1.2 times *U*_eq_ of their carrier carbon atom.

### Crystal data

**Table:**

C_19_H_13_ClO	*F*(000) = 304
*M**_r_* = 292.74	*D*_x_ = 1.291 Mg m^−^^3^
Monoclinic, *P**c*	Mo *K*α radiation, λ = 0.71073 Å
Hall symbol: P -2yc	Cell parameters from 1288 reflections
*a* = 12.0392 (14) Å	θ = 2.5–26.6°
*b* = 8.0544 (5) Å	µ = 0.25 mm^−^^1^
*c* = 7.8472 (6) Å	*T* = 295 K
β = 98.091 (10)°	Block, colourless
*V* = 753.36 (11) Å^3^	0.30 × 0.20 × 0.15 mm
*Z* = 2	

### Data collection

**Table:**

Oxford Diffraction Xcalibur (Sapphire2, large Be window) diffractometer	1866 independent reflections
Radiation source: Enhance (Mo) X-ray Source	1440 reflections with *I* > 2σ(*I*)
graphite	*R*_int_ = 0.014
Detector resolution: 8.1929 pixels mm^-1^	θ_max_ = 26.6°, θ_min_ = 2.5°
ω scan	*h* = −14→11
Absorption correction: multi-scan (*CrysAlis PRO*; Oxford Diffraction, 2009)	*k* = −9→4
*T*_min_ = 0.667, *T*_max_ = 1.000	*l* = −9→6
2481 measured reflections	

### Refinement

**Table:**

Refinement on *F*^2^	Secondary atom site location: difference Fourier map
Least-squares matrix: full	Hydrogen site location: inferred from neighbouring sites
*R*[*F*^2^ > 2σ(*F*^2^)] = 0.035	H-atom parameters constrained
*wR*(*F*^2^) = 0.083	*w* = 1/[σ^2^(*F*_o_^2^) + (0.050*P*)^2^] where *P* = (*F*_o_^2^ + 2*F*_c_^2^)/3
*S* = 0.96	(Δ/σ)_max_ = 0.001
1866 reflections	Δρ_max_ = 0.12 e Å^−^^3^
190 parameters	Δρ_min_ = −0.13 e Å^−^^3^
2 restraints	Absolute structure: Flack (1983), 423 Friedel pairs
Primary atom site location: structure-invariant direct methods	Flack parameter: 0.08 (7)

### Special details

**Table:**

Geometry. All e.s.d.'s (except the e.s.d. in the dihedral angle between two l.s. planes) are estimated using the full covariance matrix. The cell e.s.d.'s are taken into account individually in the estimation of e.s.d.'s in distances, angles and torsion angles; correlations between e.s.d.'s in cell parameters are only used when they are defined by crystal symmetry. An approximate (isotropic) treatment of cell e.s.d.'s is used for estimating e.s.d.'s involving l.s. planes.
Refinement. Refinement of *F*^2^ against ALL reflections. The weighted *R*-factor *wR* and goodness of fit *S* are based on *F*^2^, conventional *R*-factors *R* are based on *F*, with *F* set to zero for negative *F*^2^. The threshold expression of *F*^2^ > σ(*F*^2^) is used only for calculating *R*-factors(gt) *etc*. and is not relevant to the choice of reflections for refinement. *R*-factors based on *F*^2^ are statistically about twice as large as those based on *F*, and *R*- factors based on ALL data will be even larger.

### Fractional atomic coordinates and isotropic or equivalent isotropic displacement parameters (Å^2^)

**Table:**

	*x*	*y*	*z*	*U*_iso_*/*U*_eq_	
C1	0.0117 (2)	0.4299 (3)	0.5052 (3)	0.0451 (6)	
C2	−0.0556 (2)	0.5690 (3)	0.4967 (3)	0.0543 (7)	
H2	−0.0256	0.6681	0.5435	0.065*	
C3	−0.1681 (2)	0.5651 (4)	0.4195 (4)	0.0618 (7)	
H3	−0.2119	0.6605	0.4154	0.074*	
C4	−0.2124 (2)	0.4207 (4)	0.3508 (4)	0.0617 (7)	
H4	−0.2879	0.4175	0.3048	0.074*	
C5	−0.1473 (2)	0.2754 (3)	0.3471 (3)	0.0521 (6)	
C6	−0.1908 (2)	0.1292 (4)	0.2642 (4)	0.0657 (7)	
H6	−0.2661	0.1256	0.2173	0.079*	
C7	−0.1260 (3)	−0.0057 (4)	0.2513 (4)	0.0753 (9)	
H7	−0.1567	−0.1010	0.1967	0.090*	
C8	−0.0125 (3)	−0.0015 (4)	0.3204 (4)	0.0697 (8)	
H8	0.0325	−0.0934	0.3084	0.084*	
C9	0.0335 (2)	0.1362 (3)	0.4058 (4)	0.0563 (7)	
H9	0.1088	0.1355	0.4528	0.068*	
C10	−0.0321 (2)	0.2793 (3)	0.4233 (3)	0.0469 (6)	
C11	0.1246 (2)	0.4368 (3)	0.6114 (3)	0.0526 (6)	
O12	0.16532 (16)	0.3125 (2)	0.6876 (3)	0.0740 (6)	
C13	0.1853 (2)	0.5973 (3)	0.6331 (3)	0.0562 (7)	
H13	0.1563	0.6890	0.5698	0.067*	
C14	0.2804 (2)	0.6132 (3)	0.7411 (4)	0.0581 (7)	
H14	0.3076	0.5175	0.7989	0.070*	
C15	0.3469 (2)	0.7639 (4)	0.7790 (4)	0.0543 (7)	
C16	0.3072 (2)	0.9202 (3)	0.7270 (4)	0.0637 (8)	
H16	0.2357	0.9297	0.6651	0.076*	
C17	0.3705 (2)	1.0620 (4)	0.7642 (4)	0.0682 (9)	
H17	0.3421	1.1656	0.7282	0.082*	
C18	0.4770 (2)	1.0472 (4)	0.8560 (4)	0.0674 (9)	
C19	0.5195 (2)	0.8945 (4)	0.9109 (4)	0.0711 (8)	
H19	0.5912	0.8856	0.9723	0.085*	
C20	0.4540 (2)	0.7544 (4)	0.8731 (4)	0.0674 (8)	
H20	0.4821	0.6514	0.9115	0.081*	
Cl21	0.55687 (8)	1.22525 (11)	0.90310 (13)	0.1042 (4)	

### Atomic displacement parameters (Å^2^)

**Table:**

	*U*^11^	*U*^22^	*U*^33^	*U*^12^	*U*^13^	*U*^23^
C1	0.0455 (14)	0.0522 (15)	0.0381 (14)	0.0023 (11)	0.0074 (11)	0.0011 (12)
C2	0.0602 (17)	0.0538 (15)	0.0500 (16)	0.0043 (12)	0.0113 (13)	−0.0061 (13)
C3	0.0541 (17)	0.0693 (18)	0.0619 (18)	0.0165 (13)	0.0081 (14)	−0.0018 (16)
C4	0.0477 (15)	0.085 (2)	0.0521 (16)	0.0047 (14)	0.0058 (12)	0.0001 (16)
C5	0.0527 (15)	0.0656 (17)	0.0384 (15)	−0.0037 (13)	0.0074 (12)	0.0013 (14)
C6	0.0662 (18)	0.077 (2)	0.0528 (17)	−0.0111 (17)	0.0052 (14)	−0.0025 (17)
C7	0.102 (3)	0.066 (2)	0.057 (2)	−0.0178 (18)	0.0114 (18)	−0.0110 (16)
C8	0.104 (3)	0.0555 (17)	0.0522 (17)	0.0068 (16)	0.0217 (17)	−0.0032 (15)
C9	0.0653 (18)	0.0581 (15)	0.0469 (16)	0.0059 (13)	0.0126 (13)	0.0024 (14)
C10	0.0550 (16)	0.0516 (14)	0.0364 (14)	0.0008 (12)	0.0144 (12)	0.0049 (12)
C11	0.0533 (16)	0.0559 (16)	0.0491 (16)	0.0055 (12)	0.0090 (12)	0.0026 (13)
O12	0.0674 (13)	0.0677 (12)	0.0812 (15)	0.0034 (10)	−0.0100 (11)	0.0117 (12)
C13	0.0557 (17)	0.0613 (16)	0.0510 (16)	0.0029 (12)	0.0059 (14)	0.0043 (13)
C14	0.0491 (15)	0.0708 (17)	0.0544 (17)	0.0051 (13)	0.0071 (13)	0.0069 (15)
C15	0.0413 (15)	0.073 (2)	0.0478 (16)	−0.0009 (13)	0.0049 (12)	0.0011 (13)
C16	0.0448 (16)	0.079 (2)	0.065 (2)	−0.0034 (14)	−0.0015 (13)	0.0077 (16)
C17	0.0483 (17)	0.077 (2)	0.078 (2)	−0.0085 (13)	0.0042 (15)	0.0113 (16)
C18	0.0488 (18)	0.089 (2)	0.065 (2)	−0.0120 (15)	0.0101 (15)	−0.0020 (17)
C19	0.0420 (16)	0.096 (2)	0.073 (2)	0.0000 (15)	−0.0023 (14)	−0.0061 (19)
C20	0.0532 (17)	0.080 (2)	0.066 (2)	0.0088 (14)	−0.0019 (14)	0.0034 (16)
Cl21	0.0772 (5)	0.1122 (7)	0.1174 (8)	−0.0353 (5)	−0.0068 (5)	−0.0004 (6)

### Geometric parameters (Å, °)

**Table:**

C1—C2	1.379 (3)	C9—H9	0.9300
C1—C10	1.438 (3)	C11—O12	1.232 (3)
C1—C11	1.491 (3)	C11—C13	1.483 (4)
C2—C3	1.404 (4)	C13—C14	1.331 (3)
C2—H2	0.9300	C13—H13	0.9300
C3—C4	1.359 (4)	C14—C15	1.462 (4)
C3—H3	0.9300	C14—H14	0.9300
C4—C5	1.410 (4)	C15—C16	1.387 (4)
C4—H4	0.9300	C15—C20	1.395 (4)
C5—C6	1.410 (4)	C16—C17	1.382 (4)
C5—C10	1.431 (3)	C16—H16	0.9300
C6—C7	1.350 (4)	C17—C18	1.385 (4)
C6—H6	0.9300	C17—H17	0.9300
C7—C8	1.398 (4)	C18—C19	1.378 (4)
C7—H7	0.9300	C18—Cl21	1.737 (3)
C8—C9	1.371 (4)	C19—C20	1.385 (4)
C8—H8	0.9300	C19—H19	0.9300
C9—C10	1.415 (3)	C20—H20	0.9300
			
C2—C1—C10	119.2 (2)	C5—C10—C1	118.6 (2)
C2—C1—C11	118.7 (2)	O12—C11—C13	119.8 (3)
C10—C1—C11	122.0 (2)	O12—C11—C1	120.6 (2)
C1—C2—C3	121.9 (3)	C13—C11—C1	119.5 (2)
C1—C2—H2	119.1	C14—C13—C11	121.5 (3)
C3—C2—H2	119.1	C14—C13—H13	119.2
C4—C3—C2	119.4 (3)	C11—C13—H13	119.2
C4—C3—H3	120.3	C13—C14—C15	127.3 (3)
C2—C3—H3	120.3	C13—C14—H14	116.3
C3—C4—C5	122.0 (2)	C15—C14—H14	116.3
C3—C4—H4	119.0	C16—C15—C20	117.4 (3)
C5—C4—H4	119.0	C16—C15—C14	122.5 (2)
C4—C5—C6	122.1 (2)	C20—C15—C14	120.1 (3)
C4—C5—C10	118.8 (2)	C17—C16—C15	122.1 (3)
C6—C5—C10	119.1 (2)	C17—C16—H16	119.0
C7—C6—C5	121.7 (3)	C15—C16—H16	119.0
C7—C6—H6	119.1	C16—C17—C18	118.8 (3)
C5—C6—H6	119.1	C16—C17—H17	120.6
C6—C7—C8	119.7 (3)	C18—C17—H17	120.6
C6—C7—H7	120.1	C19—C18—C17	121.0 (3)
C8—C7—H7	120.1	C19—C18—Cl21	120.0 (2)
C9—C8—C7	121.0 (3)	C17—C18—Cl21	118.9 (3)
C9—C8—H8	119.5	C18—C19—C20	119.0 (3)
C7—C8—H8	119.5	C18—C19—H19	120.5
C8—C9—C10	120.9 (3)	C20—C19—H19	120.5
C8—C9—H9	119.5	C19—C20—C15	121.6 (3)
C10—C9—H9	119.5	C19—C20—H20	119.2
C9—C10—C5	117.6 (2)	C15—C20—H20	119.2
C9—C10—C1	123.7 (2)		
			
C10—C1—C2—C3	4.0 (4)	C11—C1—C10—C5	170.1 (2)
C11—C1—C2—C3	−171.1 (2)	C2—C1—C11—O12	146.6 (3)
C1—C2—C3—C4	−0.1 (4)	C10—C1—C11—O12	−28.3 (4)
C2—C3—C4—C5	−3.1 (4)	C2—C1—C11—C13	−29.9 (3)
C3—C4—C5—C6	−175.0 (3)	C10—C1—C11—C13	155.1 (2)
C3—C4—C5—C10	2.1 (4)	O12—C11—C13—C14	−3.9 (4)
C4—C5—C6—C7	175.5 (3)	C1—C11—C13—C14	172.7 (2)
C10—C5—C6—C7	−1.6 (4)	C11—C13—C14—C15	−178.0 (3)
C5—C6—C7—C8	−0.4 (4)	C13—C14—C15—C16	11.7 (4)
C6—C7—C8—C9	1.9 (4)	C13—C14—C15—C20	−169.2 (3)
C7—C8—C9—C10	−1.3 (4)	C20—C15—C16—C17	0.6 (4)
C8—C9—C10—C5	−0.7 (3)	C14—C15—C16—C17	179.7 (3)
C8—C9—C10—C1	−177.5 (2)	C15—C16—C17—C18	0.2 (4)
C4—C5—C10—C9	−175.1 (2)	C16—C17—C18—C19	−0.4 (4)
C6—C5—C10—C9	2.1 (3)	C16—C17—C18—Cl21	−180.0 (2)
C4—C5—C10—C1	1.9 (3)	C17—C18—C19—C20	−0.1 (4)
C6—C5—C10—C1	179.1 (2)	Cl21—C18—C19—C20	179.4 (2)
C2—C1—C10—C9	171.9 (2)	C18—C19—C20—C15	0.9 (4)
C11—C1—C10—C9	−13.2 (4)	C16—C15—C20—C19	−1.2 (4)
C2—C1—C10—C5	−4.8 (3)	C14—C15—C20—C19	179.7 (3)

### Hydrogen-bond geometry (Å, °)

**Table:**

*D*—H···*A*	*D*—H	H···*A*	*D*···*A*	*D*—H···*A*
C17—H17···O12^i^	0.93	2.42	3.180 (4)	139
C3—H3···Cl21^ii^	0.93	2.92	3.703 (3)	143
C8—H8···O12^iii^	0.93	2.64	3.546 (4)	163

Symmetry codes: (i) *x*, *y*+1, *z*; (ii) *x*−1, −*y*+2, *z*−1/2; (iii) *x*, −*y*, *z*−1/2.
